# Time dynamics of quantum coherence and monogamy in a non-Markovian environment

**DOI:** 10.1038/s41598-019-39027-2

**Published:** 2019-02-20

**Authors:** Chandrashekar Radhakrishnan, Po-Wen Chen, Segar Jambulingam, Tim Byrnes, Md. Manirul Ali

**Affiliations:** 1 NYU Shanghai, School of Arts and Science, 1555 Century Ave, Pudong, Shanghai, 200122 China; 2 NYU-ECNU Institute of Physics at NYU Shanghai, 3663 Zhongshan Road North, Shanghai, 200062 China; 3 Physics Division, Institute of Nuclear Energy Research, Longtan, Taoyuan, 32546 Taiwan; 4 Department of Physics, Ramakrishna Mission Vivekananda College, Mylapore, Chennai 600004 India; 5 State Key Laboratory of Precision Spectroscopy, School of Physical and Material Sciences, East China Normal University, Shanghai, 200062 China; 6 National Institute of Informatics, 2-1-2 Hitotsubashi, Chiyoda-ku, Tokyo, 101-8430 Japan; 7 Department of Physics, New York University, New York, NY 10003 USA; 8 Department of Physics, National Cheng Kung University, Tainan, 70101 Taiwan; 9 Physics Division, National Center for Theoretical Sciences, National Tsing Hua University, Hsinchu, 30013 Taiwan

## Abstract

The time evolution of the distribution and shareability of quantum coherence of a tripartite system in a non-Markovian environment is examined. The total coherence can be decomposed into various contributions, ranging from local, global bipartite and global tripartite, which characterize the type of state. We identify coherence revivals for non-Markovian systems for all the contributions of coherence. The local coherence is found to be much more robust under the environmental coupling due to an effective smaller coupling to the reservoir. This allows us to devise a characterization of a quantum state in terms of a coherence tuple on a multipartite state simply by examining various combinations of reservoir couplings. The effect of the environment on the shareability of quantum coherence, as defined using the monogamy of coherence, is investigated and found that the sign of the monogamy is a preserved quantity under the decoherence. We conjecture that the monogamy of coherence is a conserved property under local incoherent processes.

## Introduction

Coherence has been a central concept in quantum physics since the introduction of wave-particle duality. For many years the study of quantum coherence was investigated in the context of phase space distributions^1,2^ and higher order correlation functions^3^. Recently coherence was quantified in a rigorous sense by Baumgratz, Cramer, and Plenio^4^, and improved upon through several works^5–9^. In the context of these works, coherence is now viewed as a quantum characteristic alongside other quantities such as discord, entanglement, steerability, and non-local correlations^10^. It has been investigated in a variety of different systems such as Bose-Einstein condensates^11^, cavity electrodynamics^12,13^, and spin systems^14–17^.

Coherence, alongside many of the other quantum properties, are often studied without explicitly specifying the effect of the external environment on the system. Under experimentally realistic situations, the environment will cause a time varying evolution towards a mixed state^18^. A system can exhibit Markovian or non-Markovian dynamics depending on whether it is weakly or strongly coupled to the environment. Entanglement was the first type of quantum correlation whose dynamics was explored in this context^19–22^. Several studies have shown^23–26^ that the entanglement of a quantum system in a Markovian environment experiences an exponential decay with time. In a non-Markovian environment, the entanglement may reappear after a time period of complete disappearance^20,26–29^, a feature referred to as entanglement revival. Later several studies investigated the dynamics of many other quantum correlations^30–34^.

Entanglement and discord are purely inter-particle in nature, hence require at least two subsystems, but quantum coherence has the unique property^35,36^ that it can exist both at the inter-particle and intra-particle levels. The “intrinsic” or “global” coherence arises is inter-particle in nature and happens due to superposition between two qubits. Meanwhile the superposition of the quantum levels within a single subsystem results in the local coherence. These two forms of coherence have a complementary nature and cannot exceed the total coherence in the system^35–37^. Extensions of this idea can be made to multiparticle systems, in particular for a tripartite system the coherence between two subsystems limits the amount of coherence the third subsystem can share with these systems. The monogamy of coherence then measures the shareability of coherence, and is the difference between the pair-wise and the multipartite intrinsic coherences^35,36^.

In this paper, we investigate the time dynamics of the distribution of coherence under a non-Markovian environment. Our aim will be to examine the response of the various types of coherence, since as local and global, and see how susceptible they are under the incoherent operations induced by a reservoir. By changing our parameters we can equally study the Markovian limit of the environment, which will allow us to study a variety of different scenarios. We furthermore investigate the dynamics when the reservoir only partially couples to the state, on particular sites. This leads us to devise a method for understanding the nature of the coherence simply by examining the response of the total coherence by adding successive environmental couplings to the whole system. We also study the shareability of coherence by using the monogamy of coherence. This is another characteristic that occurs only for systems with at least three particles, and is a identifier of the type of correlations that are present in the system.

## Results

### Description of the model

**Figure 1:**
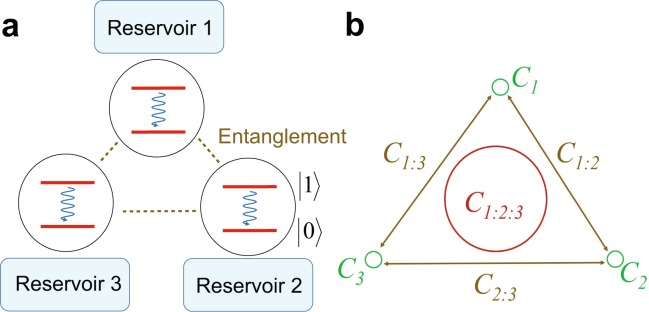
Coherence in a tripartite system. (**a**) The model examined in this paper. Three qubits are individually coupled to dissipative reservoirs which creates a decay from the $|1\rangle$ to the $|0\rangle$ state. The reservoirs can be coupled in a Markovian or non-Markovian way, depending upon the parameters of Hamiltonian (1). The initial state is generally considered to be a tripartite entangled state, of the form of a *W*, *GHZ*, or $W\bar{W}$ state. (**b**) The coherence distribution in a tripartite system. The local coherence *C*_*j*_, the bipartite global coherences *C*_*j*:*k*_, and the tripartite global coherence *C*_1:2:3_ are as marked.

We consider a system of three non-interacting parts, each consisting of a qubit interacting with a local bosonic reservoir (see Fig. 1(a)). The Hamiltonian of the qubits and reservoir reads

1 $$H=\sum _{j=1}^{3}\,[{\omega }_{0}^{j}{\sigma }_{+}^{j}{\sigma }_{-}^{j}+\sum _{k}\,{\omega }_{k}{b}_{jk}^{\dagger }{b}_{jk}+({\sigma }_{+}^{j}{B}_{j}+{\sigma }_{-}^{j}{B}_{j}^{\dagger })]$$

with ${B}_{j}={\sum }_{k}\,{g}_{k}{b}_{jk}$, where ${\sigma }_{\pm }^{j}$ are the raising and lowering operators of the two level atom and ${\omega }_{0}$ is the transition frequency of the two level system. The index *k* labels the field modes of the reservoir with frequencies ${\omega }_{k}$, ${b}_{jk}^{\dagger }$ (*b*_*jk*_) is the creation (annihilation) operator for the reservoir for the *j*th qubit, and *g*_*k*_ is the coupling strength between the qubit and the *k*th mode of the environment. This model can be solved exactly at zero-temperature^38^. The dynamics of each non-interacting part can be represented by the reduced density matrix

2 $$\rho (t)=(\begin{array}{cc}{\rho }_{11}(0)\,|h(t){|}^{2} & {\rho }_{10}(0)\,h(t)\\ {\rho }_{01}(0)\,{h}^{\ast }(t) & 1-{\rho }_{11}(0)\,|h(t){|}^{2}\end{array}),$$

where *h*(*t*) is the time evolution given by

$$\frac{{\rm{d}}h(t)}{{\rm{d}}t}=-\,{\int }_{{t}_{0}}^{t}\,d\tau f(t-\tau )h(\tau ).$$

The correlation function *f*$$(t-\tau )$$ is related to the spectral density *J*$$(\omega )$$ of the reservoir as $$f(t-\tau )=$$$$\int \,d\omega J(\omega ){e}^{i({\omega }_{0}-\omega )(t-\tau )}$$. In this work we consider a Lorentzian spectral density

3 $$J(\omega )=\frac{1}{2\pi }\frac{{\gamma }_{0}{\lambda }^{2}}{{({\omega }_{0}-\omega -{\rm{\Delta }})}^{2}+{\lambda }^{2}}.$$

for which the single qubit evolution *h*(*t*) is well known^18,19,22^

4 $$h(t)={e}^{-\frac{(\lambda -i{\rm{\Delta }})t}{2}}[\cosh (\frac{{\rm{\Omega }}t}{2})+\frac{\lambda -i{\rm{\Delta }}}{{\rm{\Omega }}}\,\sinh \,(\frac{{\rm{\Omega }}t}{2})],$$

where ${\rm{\Omega }}=\sqrt{{(\lambda -i{\rm{\Delta }})}^{2}-2{\gamma }_{0}\lambda }$. The spectral width of the reservoir *λ* characterizes the reservoir correlation time via the relation ${\tau }_{1}={\lambda }^{-1}$. Meanwhile the microscopic system-reservoir coupling *γ*_0_ is the inverse of the relaxation time ${\tau }_{2}$. The Markovian and the non-Markovian regimes can be identified from the relationship between these time scales. When ${\gamma }_{0} < \lambda /2$ (${\tau }_{2} > 2{\tau }_{1}$), the system is weakly coupled to the reservoir and the dynamics is Markovian. The non-Markovian effects due to the strong coupling regime arises when ${\gamma }_{0} > \lambda /2$ (${\tau }_{2} < 2{\tau }_{1}$).

### Local and global coherence

We investigate a non-interacting three qubit system coupled to individual bosonic reservoirs as described in the previous section. The three qubits are initialized in various states and the subsequent time evolution is examined. To characterize the different types of coherence, we use the relative entropy^4^. The total coherence in the system is given by

5 $$C(\rho )=\mathop{{\rm{\min }}}\limits_{\sigma \in  {\mathcal I} }\,S(\rho \parallel \sigma )=S({\rho }_{d})-S(\rho ),$$

where ${\mathcal I}$ is the set of incoherent state and ${\rho }_{d}$ is the diagonal matrix of the density matrix. Here ${\rho }_{d}$ is the diagonal matrix of $\rho$ in the basis $|0\rangle$ and $\mathrm{|1}\rangle$. It is only logical to investigate the process in the *σ*^*z*^-basis since the dynamics is entirely described in that basis as we notice through Eq. 2. The local coherence is then found using the relation^37^

6 $${C}_{L}=S(\pi (\rho )\parallel {[\pi (\rho )]}^{d}).$$

where $\pi (\rho )={\rho }_{1}\otimes {\rho }_{2}\otimes {\rho }_{3}$. From (5) and the (6) one can find the global coherence by simply taking the difference

7 $${C}_{G}=C-{C}_{L}.$$

In a tripartite system the global coherence can be further decomposed in to three-way and two-way global coherences. The expression for these coherences are

8 $${C}_{TG}\equiv {C}_{1:2:3}={C}_{2:3}+{C}_{1:23},$$

9 $${C}_{BG}={C}_{1:2}+{C}_{1:3}+{C}_{2:3},$$

10 $${C}_{i:j}={C}_{G}({\rho }_{ij}).$$

Here *C*_1:23_ is the intrinsic coherence between the qubit ${\rho }_{1}$ and the bipartite system ${\rho }_{23}$. If the loss of any one of the qubits completely decoheres the system then it is said to have a three-way or purely tripartite global coherence (*C*_*TG*_). Conversely if the loss of any two qubits causes complete decoherence then we have a two way or bipartite global coherence (*C*_*BG*_).

### Complete coupling with the Reservoir

In the present section we investigate tripartite system in which all the three qubits are coupled to the environment. For the initial state we use the $W\bar{W}$-state defined as

11 $$|W\bar{W}\rangle =\frac{|W\rangle +|\bar{W}\rangle }{\sqrt{2}}$$

where

12 $$\begin{array}{l}|W\rangle =[|001\rangle +|010\rangle +|100\rangle ]/\sqrt{3}\\ |\bar{W}\rangle =[|011\rangle +|101\rangle +|110\rangle ]/\sqrt{3}.\end{array}$$

This is particularly interesting as it has all types of different coherences, both local and global, distributed in both a tripartite and bipartite manner. This is in contrast to either *GHZ* or *W* states, which have zero local coherence, and are purely tripartite and bipartite entangled^35^. By examining a state with all types of coherences this gives a convenient way of examining the time dynamics of the various contributions.

**Figure 2:**
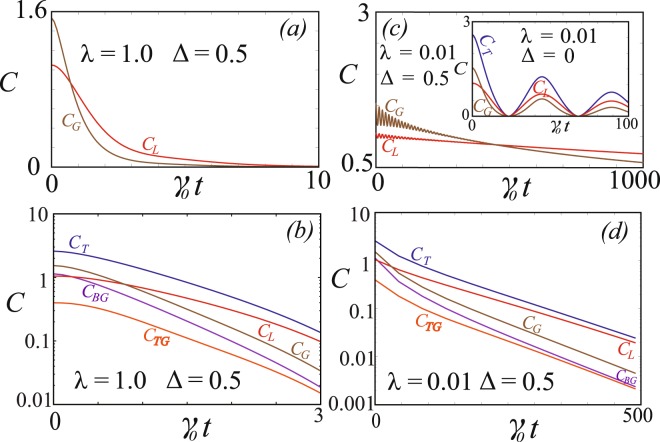
Time dynamics of coherence for the $W\bar{W}$ state. (**a**) The local coherence *C*_*L*_ and global coherence *C*_*G*_ in the Markovian regime. Parameters are *λ* = 1, ${\rm{\Delta }}=0.5$. (**b**) Semi-logarithmic plot for the same parameters as (**a**), including additionally the total coherence *C*_*T*_, bipartite coherence *C*_*BG*_, tripartite coherence *C*_*TG*_. (**c**) Various coherences as marked in the non-Markovian regime. Parameters are *λ* = 0.01 and ${\rm{\Delta }}=0.5$ (main figure), ${\rm{\Delta }}=0$ (inset). (**d**) Semi-logarithmic plot for the non-Markovian regime for the same parameters as the main plot of (**c**) for the envelope function of the coherences as marked.

The dynamics of the $W\bar{W}$ state in the Markovian regime is illustrated in Fig. 2(a). We see that the local and global coherence exhibits exponential decay but at different rates. To find the decay rate we plot the coherence on a semi-logarithmic plot as a function of the dimensionless time *γ*_0_*t* as shown in Fig. 2(b). The gradient of the curve on the semi-logarithmic plot gives the decay rate at a particular time. We find that at any given time the local coherence has a lower decay rate than the global coherence. This is the expected result since by its very nature, local coherence is only present at each qubit site, which is coupled locally to the reservoir. Thus the local coherence only experiences effectively one reservoir at a time, whereas the global coherence is distributed across the whole system. It is thus affected by all reservoirs at the same time, and should therefore decay at a faster rate.

We now consider the strongly non-Markovian limit, which gives richer dynamics to all types of coherence. The non-Markovian dynamics of the $W\bar{W}$ state both with detuning and without detuning is given in Fig. 2(c). From the plots we observe that both the local and global coherence have different dynamical behavior. In particular we see the decay and revival of the coherence in an analogous way to that observed with entanglement^20,26^. We can accordingly call this phenomena “coherence revival”, since coherence spontaneously re-enters the system after being initially destroyed by the bath. For the case with zero detuning the coherence in fact is completely destroyed in all forms, and then spontaneously reappears. The general phenomenology of the coherence in the non-Markovian regime is that it oscillates with a decaying envelope. Thus the quantum coherence which is oscillatory at shorter time scale has an exponential decay at the longer time scale. To find the decay rate we trace this exponential envelope by performing a linear fit of the logarithm of the coherence at the maximum point as a function time. The slope of the semi-logarithmic plots in Fig. 2(d) then give the decay rate for the non-Markovian case. Here too we find that the local coherence has a slower decay compared with the global coherence, due to only a single reservoir acting on the local coherence, in comparison to multiple reservoirs acting on global coherence.

In Fig. 2(b,d) we compare the decay rate of the local coherence with the total bipartite global coherence *C*_*BG*_ and the tripartite global coherence *C*_*TG*_. From the linear fit we observe that the local coherence has the lowest decay rate. Further we notice that the total coherence has a lower decay rate compared with any one of its individual components. Also the total global coherence has a lower decay rate compared with the bipartite and the tripartite global coherence. Both these observations are along the expected lines since the individual components have a faster decay rate than their combined value.

**Figure 3:**
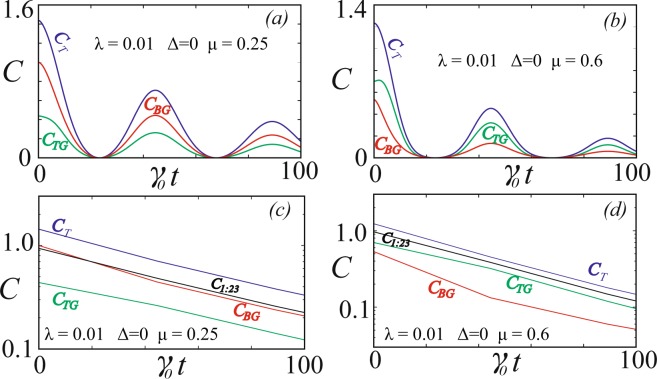
Time dynamics of quantum coherence for the superposition of GHZ and W states. (**a**) The total coherence *C*_*T*_, the bipartite global coherence *C*_*BG*_ and the tripartite global coherence *C*_*TG*_ in the non-Markovian regime. Parameters are *μ* = 0.25, *λ* = 0.01 and ${\rm{\Delta }}=0$. (**b**) The total coherence *C*_*T*_, the bipartite global coherence *C*_*BG*_ and the tripartite global coherence *C*_*TG*_ in the non-Markovian regime. Parameters are *μ* = 0.60, *λ* = 0.01 and ${\rm{\Delta }}=0$. (**c**) Semi-logarithmic plot for the envelope of the same parameters as in (**a**). (**d**) Semi-logarithmic plot for the non-Markovian regime for the envelope of the same parameters as in (**b**).

It is well known that the total coherence in a GHZ state is entirely tripartite in nature, whereas in a W-state the coherence is distributed in a bipartite manner. Another example of a state which possesses different types of coherences is the linear superposition of GHZ and W states introduced in^39^ and defined as

13 $$|GW\rangle =\mu |GHZ\rangle +(1-\mu )|W\rangle $$

which exhibits the crossover of bipartite and tripartite coherences. In Fig. 3 the non-Markovian evolution of quantum coherence is described for two instances namely (a) $\mu =0.25$ where the W state dominates over the GHZ state and (b) $\mu =0.60$ where the GHZ state contributes more to the superposition. From the plots Fig. 3(a) we can see that the bipartite global coherence is more dominant for $\mu =0.25$ because the W-state contributes more to the superposition. In the case of $\mu =0.6$ the tripartite global coherence is higher since the GHZ state has a higher contribution to the superposition. In Fig. 3(c,d) we compare the decay rate of the total, bipartite global and the tripartite global coherence of both the plots in Fig. 3(a,b). In both the situations $\mu =0.25$ and $\mu =0.6$ the bipartite global coherence has a lower decay rate compared with the tripartite global coherence. The total coherence has a lower decay rate compared with both the bipartite global coherence and tripartite global coherence which is along the expected lines.

### Partial coupling to the reservoir

In the investigations so far described, all three qubits were connected to an external reservoir. Now we would like to examine the situation where only some of the qubits are connected to the reservoir. This can be achieved by changing the decay time of the couplings on the different qubits. For example, the single channel decay regime can be defined as when the decay time of qubit 1 is much less than the remaining qubits and also the observation time scale $\tau$

14 $${t}_{1} < \tau \ll {t}_{2,3}$$

where *t*_*j*_ is the decay time of the *j*th qubit.

The states that we will examine here are the *W* and the *GHZ* states, defined as

15 $$|GHZ\rangle =[|000\rangle +|111\rangle ]/\sqrt{2}.$$

These are known to have a different structure of entanglement, and therefore its coherence properties can be expected to be different. The *GHZ* state is considered to be a genuinely tripartite entangled system, whereas the *W* state is bipartite entangled, and are unrelated under local operations and classical communications. By changing the local couplings to the reservoir, it is reasonable to expect that these respond differently given the considerations of the previous section.

**Figure 4:**
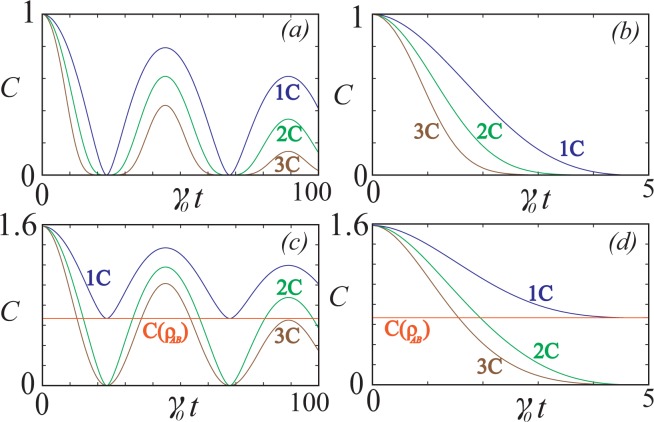
Time dynamics of coherence for the *GHZ* and *W* states, for various reservoir couplings. (**a**) Non-Markovian evolution for the *GHZ* state for the number of reservoir couplings as marked. Parameters used are *λ* = 0.01 and ${\rm{\Delta }}=0$. (**b**) Markovian evolution with parameters used *λ* = 1.0 and ${\rm{\Delta }}=0$. (**c**) Non-Markovian evolution for the *W* state. Parameters used are *λ* = 0.01 and ${\rm{\Delta }}=0$. (**d**) Markovian evolution with parameters used *λ* = 1.0 and ${\rm{\Delta }}=0$.

In Fig. 4(a,b), we show the variation of quantum coherence for the one, two, and three channels for both the Markovian and the non-Markovian regimes with an initial *GHZ* state. We find that under the Markovian approximation the quantum coherence always vanishes to zero in the long-time limit. For the non-Markovian case we observe that the quantum coherence oscillates with time on the shorter time scale, while decaying in the long-time limit. The single channel coherence oscillations decays slower and rises faster in comparison with the two and the three channel cases. In the three channel case the coherence falls to zero and remains so for a particular length of time. This behavior is because in the single channel only one qubit is directly in contact with the environment and the other two qubits are influenced by the environment due to their coherent connection with the first one.

The behavior of *W* states is shown in Fig. 4(c,d) for the Markovian and the non-Markovian cases respectively. From the plots we observe that the three and the two channel coherence goes to zero but the single channel case attains a steady state value of *C* = 2/3 for the Markovian case. In the non-Markovian case the quantum coherence oscillates, but the oscillatory minimum is zero for the two and three channel cases but for the single channel case it is equal to *C* = 2/3. This is because the coherence in the *W* state is distributed in a bipartite manner and the environment is acting on a single qubit in the single channel case. In the long-time limit the *W* state evolves to the mixed state

16 $$\begin{array}{rcl}{\rho }_{1{\rm{C}}}(t\to \infty ) & = & \sum _{i}\,{M}_{i}|W\rangle \langle W|{M}_{i}^{\dagger }\\  & = & |0\rangle \langle 0|\otimes [\tfrac{2}{3}(\tfrac{|01\rangle +|10\rangle }{\sqrt{2}})\,(\tfrac{\langle 01|+\langle 10|}{\sqrt{2}})+\tfrac{1}{3}|00\rangle \langle 00|]\end{array}$$

where we have taken the measurement operators on the first qubit

17 $$\begin{array}{rcl}{M}_{0} & = & |0\rangle \langle 0|\\ {M}_{1} & = & |0\rangle \langle 1|\end{array}$$

corresponding to a decay of a qubit. As we can see from (16) the decoherence of the single qubit does not completely destroy the total coherence in the system. The value *C* = 2/3 attained in the steady state limit for the Markovian situation and as the oscillatory minimum in the non-Markovian situation is the coherence between the two qubits which are not influenced by the environment in any way. The value *C* = 2/3 correspond to the probability of the Bell state in (16), which is the only contribution to the coherence in this case. For the two and three channel coherences, it is easy to see that further measurement of (16) will completely collapse the Bell state superposition, hence eventually there is zero coherence in the long-time limit. We note that this is in stark contrast to the coherence in a *GHZ* state which is distributed in a completely tripartite manner such that the decoherence of a single qubit will always destroy the total coherence in the system.

From the above results we observe that by coupling the reservoir in different ways, information can be obtained about how coherence is distributed in a multipartite system. For tripartite systems the quantum coherence can be characterized in the manner shown in Fig. 1(b). Firstly, there are three local coherences, one for each qubit. Next, there are the three bipartite global coherences *C*_1:2_, *C*_1:3_ and *C*_2:3_, according to the pairings of each qubit. Lastly, there is a genuinely tripartite global coherence *C*_1:2:3_. The total coherence can be distributed in only these seven different contributions. Hence we can construct a seven-tuple

18 $${\mathscr{C}}=\{{C}_{1},{C}_{2},{C}_{3},{C}_{1:2},{C}_{1:3},{C}_{2:3},{C}_{1:2:3}\}$$

which contains all the information about the distribution of coherence in the system. Clearly the above procedure can be generalized in an analogous way for a multipartite system. If we do not have a knowledge of an initial quantum state, we can reverse engineer the state if we have the time evolution dynamics of the quantum coherence which can provide us with this seven-tuple. For example, by coupling a reservoir to qubit 1 and looking at the steady state, the coherence with all terms involving qubit 1 will be destroyed, yielding

19 $${\mathscr{C}}{|}_{{{\rm{\Pi }}}_{1}}=\{0,{C}_{2},{C}_{3},0,0,{C}_{2:3},0\},$$

where ${{\rm{\Pi }}}_{j}$ denotes a measurement on the *j*th qubit. Similarly the coherence involving qubit 2 can be destroyed by applying two reservoirs

20 $${\mathscr{C}}{|}_{{{\rm{\Pi }}}_{1}{{\rm{\Pi }}}_{2}}=\{0,0,{C}_{3},0,0,0,0\},$$

which yields the local coherence on qubit 3 alone. By combining all possible measurement combinations *I*, ${{\rm{\Pi }}}_{1}$, ${{\rm{\Pi }}}_{2}$, ${{\rm{\Pi }}}_{3}$, ${{\rm{\Pi }}}_{1}{{\rm{\Pi }}}_{2}$, ${{\rm{\Pi }}}_{1}{{\rm{\Pi }}}_{3}$, ${{\rm{\Pi }}}_{2}{{\rm{\Pi }}}_{3}$ we can deduce all the coherences within the system. The number of possible measurement combinations is always guaranteed to be the same as the number of different coherences because the coherences appear as all *n*-way groupings of the subsystems, which is the same as for the measurements. This allows a consistent evaluation of coherence in any multipartite system.

### Monogamy of coherence

We have seen that in a multipartite system the global coherence can be further decomposed into the bipartite contribution, tripartite contribution and so on up to the *N*-partite contributions^35,37^. Quantum systems thus have a unique way sharing coherence which is captured by the monogamy of coherence introduced in ref.^35^, in analogy with the monogamy of entanglement^40,41^. For a tripartite system the monogamy of coherence reads:

21 $$M={C}_{1:2}+{C}_{1:3}-{C}_{1:23}$$

Here *C*_1:2_ (*C*_1:3_) denotes the global coherence between the qubits 1 & 2 (1 & 3) and *C*_1:23_ is the global coherence between qubit 1 and the bipartite block 23. In a genuinely tripartite coherent system, the system is described as being monogamous and we observe *M* ≤ 0. When *M* > 0, the bipartite coherence is more dominant and the system is polygamous. *GHZ* and *W* states are archetypal examples of a polygamous and monogamous state respectively.

**Figure 5:**
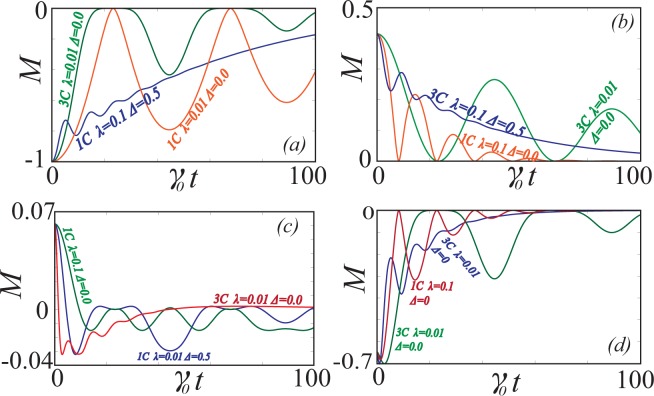
The time evolution of the monogamy of coherence for (**a**) *GHZ*, (**b**) *W* states and a quantum state which is a linear superposition of GHZ and W state for (**c**) *μ* = 0.25 and (**d**) *μ* = 0.60, under various conditions as labeled.

In Fig. 5 we calculate the time evolution of the monogamy of coherence of the *GHZ* and *W* states under various non-Markovian conditions. In all cases that we have calculated we observe that the monogamy of coherence does not change sign, and retains its initial character. In the strongly non-Markovian regime, the monogamy of coherence can become zero, particularly at points where the overall coherence, and hence its constituents vanish

22 $${C}_{1:2}={C}_{1:3}={C}_{1:23}=0.$$

We have verified that there is no violation of the sign preservation by examining points where *M* is small and have not found any exceptions. This can be understood to be due to the fact that the quantum symmetries of the system regarding the spatial distribution do not change under time evolution. The form of the reservoir coupling is strictly in a local fashion, and in a general sense corresponds to a local operation. Since the *GHZ* and *W* states are known to be either polygamous and monogamous under local operations^35^, and our reservoir model falls under the same category of operations. This means that *M* correctly characterizes the polygamous or monogamous nature of the state. We thus conjecture that a quantum state under a local time evolution process will preserve the monogamy of coherence in a multipartite system.

The time evolution of monogamy of quantum coherence for the state $|GW\rangle$ is discussed in Fig. 5(c,d) under different non-Markovian conditions. The case of *μ* = 0.25 is given through Fig. 5(c) where the dynamics of shareability of coherence is discussed. We find that the state oscillates between monogamy and polygamy throughout the decay. In general the states are polygamous for $\mu \lesssim 0.20$ and it exhibits both monogamous and polygamous nature in the region $0.20\lesssim \mu \lesssim 0.35$ and completely monogamous when $\mu \gtrsim 0.35$. The major reason is that the coherence between the bipartite partition between the qubit 1 and the joint system 23 namely *C*_1:23_ decays much slower than the total bipartite global coherence in the system which can be observed from Fig. 3(c). Hence the system is monogamous for most of the time though polygamous nature appears at few instances. In the case of *μ* = 0.6 from Fig. 5(d) we find that the state is monogamous through the entire evolution process.

## Discussion

The time evolution of quantum coherence of a three qubit system each interacting with a local environment was been investigated in both the Markovian and non-Markovian limits. For the tripartite $W\bar{W}$ system the total coherence can be decomposed into the local and the global components, according to whether the coherence are intra- or inter-qubit in nature. Due to the tripartite nature of the system, the global coherence can be decomposed into the bipartite global coherence and the tripartite global coherence. In analogy to entanglement revivals, we observed coherence revivals in the non-Markovian case, where the coherence can return to the system from the reservoir. In several cases this was observed to occur even after the coherence collapsed to zero. The general observation from this is that the local coherence decays much slower in comparison with other forms of coherence. This behavior is irrespective of whether the dynamics is Markovian or non-Markovian. This points to the fact that local coherence is much more robust in the presence of decoherence than global coherence. Previous studies^42–44^ have shown that the robustness varies with the nature of a quantum state. Contrast to these works we show that the different contributions of coherence decay at different rates in the same quantum state. Therefore, localizing the coherence can be one effective strategy towards extending the life-time of a quantum state in physical systems. By temporarily storing it in this form, and converting it to global coherence according to the complementary nature of coherence, this can be an effective strategy towards preserving coherence in the system. It is interesting to note that recently experiments have been carried out in which the interconversion of quantum coherence in to other quantum correlations like discord and entanglement have been the main focus of the study. Particularly in^45,46^ the local coherence in a quantum system has been converted into discord which was again successfully steered into local coherence. This experiment establishes the feasibility of interconversion of local and global coherence which may help us to prolong the quantum correlations in a system. Further it has been shown that local resources are relevant for applications in quantum metrology^47^ and it has been shown that in some distributed quantum computation protocols, global coherence provides the necessary resource for a computational speed up compared to classical algorithms^48,49^. Hence a understanding of the dynamics of the different forms of coherence may be useful to choose the suitable quantum states for the relevant practical applications.

We also investigated the response of the system to changing the number of reservoir couplings throughout the system. For both the *GHZ* and *W* states, the three channel coherence decayed the state more rapidly in comparison with the single channel coherence. By changing the number of reservoir couplings to the system, it was found that various types of coherence could be selectively destroyed, giving a characterization of the coherence in the system. For example, with only one reservoir coupled to the system, the coherence does not go to zero for *W* state but it does so for a *GHZ* state. This is due to the way in which coherence is distributed among the qubits. This leads us to the characterization of the coherence in a multipartite system according to (18). By coupling reservoirs in various configurations one can selectively “turn off” the coherence for various contributions. Since the number of ways of reservoir couplings is always guaranteed to be the same as the number of elements in the coherence tuple, this allows a powerful way of characterizing the coherence in a multipartite system. Finally from the time evolution of the monogamy of coherence we found that the system preserves it initial nature of either monogamy or polygamy. This can be explained due to the local couplings of the reservoirs, which can be viewed as incoherent local operations on the system. Such operations are known not to change the character of the system in term of monogamy or polygamy in the context of entanglement. We find that this is true also in the coherence case, and conjecture that the sign of the monogamy of coherence is a preserved quantity for local operations. We found no numerical violations to this, for all the parameters and states that were tried.

The extension of coherence to non-unitary evolution has shown that we can obtain several interesting characterizations of the original quantum state. The rate of decay of the various coherences gives the robustness of the state under environmental influence. By examining its distribution one can directly observe that the state evolves in such a way that certain components of its coherence decay faster than others. Thus under partial decoherence one might expect to find that the more robust types of coherence are predominantly left. By looking in the long-time limit one can even completely characterize the distribution of the coherence. Remarkably, the number of measurement combinations is equal to the number of coherences, which show that this is always possible using this prescription. One possible extension of our work is to look at more complex systems, which can be used as a method of characterizing many-body quantum states. This program has already been started in several works such as refs^14–17^ and could be used in contexts such as detecting quantum phase transitions.
